# Use of Antihistamine Drugs in Colitis: A Review

**DOI:** 10.3390/ph19071124

**Published:** 2026-07-21

**Authors:** Bartosz Bogielski, Dariusz Gach, Katarzyna Michalczyk, Bronisława Skrzep-Poloczek, Mateusz Stojko, Jolanta Zalejska-Fiolka, Dominika Stygar

**Affiliations:** 1 Branch in Bielsko-Biała, Medical University of Silesia, 40-055 Katowice, Poland; bartosz.bogielski@sum.edu.pl (B.B.); katarzyna.michalczyk@sum.edu.pl (K.M.); jzalejskafiolka@sum.edu.pl (J.Z.-F.); dstygar@sum.edu.pl (D.S.); 2 Scientific Research Facility, Branch in Bielsko-Biała, Medical University of Silesia, 40-055 Katowice, Poland; dariusz.gach@sum.edu.pl (D.G.); mateusz.stojko@sum.edu.pl (M.S.); 3 Department of Physiology, Faculty of Medical Sciences in Zabrze, Medical University of Silesia, 40-055 Katowice, Poland; 4 Department of Biochemistry, Faculty of Medical Sciences in Zabrze, Medical University of Silesia, 40-055 Katowice, Poland

**Keywords:** colitis, histamine receptor antagonists, oxidative stress markers

## Abstract

**Background/Objectives**: Colitis involves both local intestinal damage and systemic dysfunction, driven by oxidative stress and immune imbalance. Histamine, acting through its receptors, influences these processes, yet its therapeutic relevance in colitis remains unclear. This review systematically examines histamine-mediated signaling in colitic inflammation and evaluates preclinical and clinical data on antihistamines to assess their potential as a mechanism-based treatment. **Methods**: A structured literature search was conducted using PubMed and Google Scholar to identify studies addressing histamine signaling and the use of antihistamines in colitis, using keywords such as “colitis,” “histamine,” “histamine receptors,” and “antihistamines.” Relevant experimental and clinical studies were screened and critically analyzed to provide an integrated overview of mechanistic and therapeutic insights. This review was designed as a mechanistic narrative synthesis based on a structured search and qualitative appraisal rather than a formal systematic review with quantitative risk-of-bias grading. **Results**: Findings from preclinical investigations consistently indicate that pharmacological manipulation of histamine signaling—especially through H3 and H4 receptor subtypes—reduces inflammatory activity and modulates oxidative balance in animal models of colitis. Conversely, clinical evidence concerning H1 and H2 receptor antagonists remains scarce and discordant, lacking sufficient support for a definitive causal relationship or unambiguous therapeutic efficacy. Importantly, no clinical studies to date have assessed the effects of selective H3 or H4 receptor blockers in individuals with colitis, revealing a substantial disconnect between bench research and bedside application. Existing human trials have mainly concentrated on mucosal endpoints, with little attention paid to systemic manifestations or oxidative stress-related parameters. **Conclusions**: Histamine-dependent signaling constitutes a mechanistically credible target in colitis, connecting immune activation, impairment of the epithelial barrier, and oxidative injury. Although experimental data are encouraging, clinical corroboration remains absent. Antihistamines may hold greater promise for alleviating systemic oxidative stress than for directly ameliorating intestinal inflammation. Rigorously designed clinical trials that incorporate both gastrointestinal and systemic outcome measures are necessary to elucidate their therapeutic position.

## 1. Introduction

Colitis, including ulcerative colitis and Crohn’s disease, is a highly prevalent chronic inflammatory disorder affecting millions of individuals worldwide. The incidence and prevalence of inflammatory bowel diseases continue to increase globally, imposing a substantial healthcare burden and significantly reducing patients’ quality of life. In addition to gastrointestinal symptoms, colitis is associated with numerous extra-intestinal manifestations affecting multiple organs and systems [1,2,3,4,5,6,7].

The pathogenesis of colitis is complex and involves interactions between genetic susceptibility, environmental factors, intestinal microbiota, and dysregulated immune responses. Excessive production of reactive oxygen species (ROS) constitutes an important pathogenic mechanism contributing to epithelial barrier disruption, activation of inflammatory pathways, and progressive tissue injury [8,9,10].

Histamine, a biologically active amine with pleiotropic immunomodulatory functions, plays a central role in inflammatory signaling and may contribute to redox imbalance through receptor-mediated activation of immune cells and downstream oxidative pathways [11]. Given the established link between oxidative stress and colitis, as well as the capacity of histamine to modulate inflammatory and oxidative responses, a mechanistic relationship between histamine signaling and the systemic consequences of colitis warrants careful evaluation [12,13,14,15,16,17,18]. Despite increasing evidence implicating histamine and its receptors in intestinal inflammation, a comprehensive synthesis of experimental and clinical data regarding antihistaminic therapies in colitis remains lacking. Therefore, in the present review, we comprehensively examined current knowledge regarding histamine signaling, histamine receptor subtypes, and the potential therapeutic role of antihistaminic agents in colitis.

## 2. Materials and Methods

The content of this review was developed through a structured search of the PubMed and Google Scholar databases to identify and analyze the existing literature on the role of histamine signaling and the use of antihistamine drugs in colitis. A comprehensive search strategy was applied using a combination of relevant keywords, including “colitis”, “inflammatory bowel disease”, “histamine”, “histamine receptors”, “H1 receptor”, “H2 receptor”, “H3 receptor”, “H4 receptor”, “antihistamines”, “oxidative stress”, “intestinal permeability”, and “immune response.” These terms were used both individually and in various combinations to ensure broad yet focused coverage of the topic.

The search strategy was designed to include original research articles as well as review papers investigating mechanistic, experimental, and clinical aspects of histamine signaling in colitis. Both animal and human studies were considered to provide a comprehensive translational perspective. To ensure relevance and consistency, only articles published in English were included. The latest search was done on 27 March 2026.

Following the initial search and duplicate removal, studies were screened based on titles and abstracts by three independent researchers to assess their relevance to the objectives of this review. Articles that did not address histamine-related mechanisms, antihistamine therapy, or inflammatory processes in colitis were excluded. The remaining studies (*n* = 299) underwent full-text evaluation, during which additional exclusions were made based on insufficient methodological quality, lack of relevance, or absence of mechanistic or clinical insight.

The selected articles (*n* = 240) were subsequently critically analyzed and synthesized to provide an integrated overview of current knowledge regarding the role of histamine signaling and the potential therapeutic application of antihistamine agents in colitis. This review follows a mechanistic, translational narrative approach based on a structured literature search, rather than a formal systematic review or meta-analysis. Consequently, authors did not apply standardized study-by-study risk-of-bias tools (such as the SYRCLE animal research scale, the Newcastle–Ottawa Scale for cohort studies, or randomized-trial risk-of-bias instruments) and instead qualitatively considered study design, sample size, model type, and translational relevance when interpreting individual findings.

## 3. Colitis

Colitis is defined as inflammation of the colonic mucosa, characterized by structural and functional alterations of the large intestine [19]. It encompasses a heterogeneous spectrum of disorders with diverse etiologies, including immune-mediated mechanisms, genetic susceptibility, infectious, ischemic, drug-induced, and idiopathic causes [1,2,3,4,5,6,7]. Clinically, colitis typically presents with diarrhea, abdominal pain, urgency, tenesmus, and, in more severe cases, rectal bleeding accompanied by systemic inflammatory manifestations [20,21].

The disease course is frequently chronic or relapsing–remitting, reflecting a complex interplay between mucosal immune dysregulation, epithelial barrier dysfunction, gut microbiota alterations, and inflammatory mediators such as cytokines, eicosanoids, and biogenic amines, including histamine [22,23,24,25].

Given its multifactorial pathophysiology, effective management requires a holistic and integrative therapeutic approach extending beyond symptomatic control. Current strategies combine dietary modulation, pharmacological anti-inflammatory and immunomodulatory agents, microbiota-targeted interventions, and supportive care [26,27,28]. Nevertheless, therapeutic limitations and incomplete disease control in a substantial proportion of patients underscore the need for novel or adjunctive treatment strategies targeting specific inflammatory pathways.

Inflammatory bowel disease (IBD) encompasses a spectrum of chronic, immune-mediated conditions, with ulcerative colitis (UC) and Crohn’s disease (CD) representing its two primary forms [29,30,31]. These entities differ fundamentally in anatomical distribution, depth of inflammation, histological features, and associated complications. Ulcerative colitis is limited to the colon and rectum, where it manifests as continuous, superficial mucosal inflammation that typically begins in the rectum and extends proximally in an uninterrupted pattern [21,32]. In contrast, Crohn’s disease can involve any part of the gastrointestinal tract—from the oral cavity to the perianal area—and is characterized by discontinuous “skip” lesions and transmural involvement, which predispose patients to complications such as strictures, fistulas, and penetrating disease [33]. Persistent activation of mucosal immune pathways, accompanied by oxidative stress and impaired epithelial barrier integrity, promotes both intestinal inflammation and systemic consequences, further emphasizing the multifactorial and socially relevant nature of IBD [34,35,36]. Despite overlapping immunological and clinical characteristics, these distinct pathological and anatomical features have critical implications for disease progression, therapeutic decision-making, and long-term outcomes.

Inflammatory bowel disease (IBD), comprising ulcerative colitis and Crohn’s disease, represents a growing global health concern [37]. Although historically most prevalent in North America and Western Europe, its incidence is rapidly increasing in newly industrialized regions across Asia, South America, and the Middle East, a trend closely associated with urbanization and Westernized lifestyle factors and habits [38]. Dietary patterns rich in processed foods, saturated fats, refined sugars, and food additives have been linked to intestinal inflammation and microbiota dysbiosis [39]. Early-life antibiotic exposure further contributes to altered microbial composition and increased disease susceptibility, particularly in pediatric populations [40].

IBD typically manifests in early adulthood; however, pediatric-onset disease is increasingly recognized [41,42]. Sex distribution varies by subtype, with Crohn’s disease often demonstrating a slight female predominance, whereas ulcerative colitis may show male predominance or a balanced distribution depending on the population studied—cohort included 410 patients with Crohn’s disease (51% female) and 483 individuals with Ulcerative colitis (56% male) [37,43]. These epidemiological differences likely reflect interactions among hormonal influences, immune regulation, genetic predisposition, and environmental exposures.

Beyond its evolving epidemiology, IBD imposes a substantial clinical and socioeconomic burden. High direct healthcare costs, frequent hospitalizations, impaired quality of life, reduced work productivity, and the need for long-term surveillance characterize the disease [44,45,46]. Its persistent pattern of flare-ups and remissions necessitates ongoing therapy and, in a significant proportion of patients, surgical intervention [47]. Complications like strictures, dysplastic changes, and colorectal cancer heighten the associated morbidity [48]. Collectively, these features establish IBD as a chronic, high-impact inflammatory disorder requiring improved and more precisely targeted therapeutic approaches.

## 4. Systemic Consequences of Colitis: A Histaminergic Perspective

Although colitis is fundamentally characterized by inflammation of the colonic mucosa, its biological and clinical impact extends well beyond the gastrointestinal tract [49,50,51]. This is especially evident in inflammatory bowel disease (IBD), where intestinal inflammation is now recognized as a manifestation of broader systemic immune dysregulation rather than a process confined solely to the colon [52]. As the gut serves as a key immunological interface between the host and the external environment, disruption of mucosal homeostasis can precipitate widespread immune activation with significant systemic consequences [35,53].

Chronic colonic inflammation is associated with increased activation of mucosal mast cells and enhanced histamine release within the intestinal mucosa. Human studies have shown that mucosal histamine content and histamine secretion are significantly increased in affected colonic tissue from patients with Crohn’s disease and ulcerative colitis compared with unaffected tissue or healthy controls [54,55]. In addition, urinary excretion of N-methylhistamine, a stable histamine metabolite, was elevated in patients with active Crohn’s disease (7.1 ± 4.2) and active ulcerative colitis (8.1 ± 4.8) compared with controls (4.6 ± 1.9), and correlated with clinical disease activity [56]. However, plasma histamine concentrations may remain within the normal range in many patients with IBD, indicating that histamine-mediated processes are more consistently detectable at the mucosal or metabolite level than in systemic plasma measurements. Under physiological conditions, histamine participates in mucosal defense and immune surveillance; however, in the context of colitis, dysregulated histamine release may extend its effects beyond the intestinal compartment [57,58,59,60].

### 4.1. Hepatobiliary Disorders

Several experimental studies have demonstrated that chemically induced colitis leads to significant oxidative damage in hepatic tissue, including elevated malondialdehyde (MDA) levels and reduced total antioxidant capacity (TAC), indicating a strong gut–liver axis mediated by oxidative mechanisms. For example, a study using the dextran sodium sulfate (DSS)-induced colitis model in mice showed marked hepatic oxidative stress and inflammation, which were mitigated by increased endogenous n-3 polyunsaturated fatty acids (PUFAs) [61]. Similarly, Yao et al. [62] demonstrated that colitis-induced liver injury involved oxidative and inflammatory pathways, which could be alleviated by treatment with 2′-fucosyllactose, suggesting a functional gut-liver-microbiota interaction [62]. Furthermore, clinical and experimental reviews underscore that oxidative and nitrosative stress not only exacerbate intestinal mucosal injury in inflammatory bowel disease (IBD), but also contribute to extraintestinal manifestations such as hepatobiliary complications [63]. Increased reactive oxygen species (ROS) generation, lipid peroxidation, and antioxidant depletion in hepatic tissue have been documented in IBD models and patients [64,65,66,67]. Experimental colitis models (e.g., DSS-induced colitis) consistently show elevated ROS levels alongside reductions in endogenous antioxidants such as glutathione and superoxide dismutase [8]. Clinical studies further reveal increased levels of malondialdehyde (MDA)—a key lipid peroxidation marker—in the plasma of Crohn’s disease and ulcerative colitis patients compared to healthy controls [9]. Systematic review underlines the prevalence of oxidative stress in IBD, evidenced by widespread alterations in both oxidative damage markers and antioxidant defenses [10]. Emerging evidence suggests that histamine signaling may contribute to the pathogenesis of immune-mediated liver injury, offering potential therapeutic targets. The liver expresses all four histamine receptor subtypes (H1R-H4R), with H1R and H4R appearing particularly relevant to inflammatory processes [68,69,70,71]. There are scientific reports indicating that modulation of histamine levels influences the progression of hepatopathy. Shen et al. reported that H2 receptor antagonist use was associated with a lower prevalence of non-alcoholic fatty liver disease (NAFLD), suggesting a potential role of histaminergic signaling in hepatic metabolic and inflammatory processes. Although these findings are not specific to colitis-associated liver disease, they support the concept that histamine receptor modulation may influence extra-intestinal hepatic manifestations [72].

### 4.2. Neurological Disorders

Colitis, as a part of IBD, has increasingly been associated with neurological and neuropsychiatric disorders, reflecting the complex bidirectional gut–brain axis communication, including influence on the central and peripheral nervous systems [73,74,75,76,77].

Individuals with chronic colitis show an increased prevalence of neuropsychiatric symptoms, including anxiety, depression, cognitive dysfunction, and persistent fatigue [78]. Moreover, emerging research points to potential associations between sustained colonic inflammation and neuroinflammatory or neurodegenerative disorders, possibly driven by systemic cytokine release, increased intestinal permeability, and microbial translocation [79].

Compromised gut barrier and persistent mucosal immune activation promote excessive generation of ROS and pro-inflammatory cytokines, which enter the circulation and eventually impair blood–brain barrier integrity and function and modulate immune activity within the central nervous system [80,81,82]. Increased intestinal permeability further facilitates microbial translocation, amplifying systemic inflammatory signaling. Emerging evidence suggests that these mechanisms may link chronic colitis with neuroinflammatory and neurodegenerative disorders [83,84,85]. Among the mediators potentially involved in this axis, histamine represents a biologically relevant candidate, given its role in immune modulation, oxidative processes, and neuroimmune communication [86,87]. Beyond its local actions in the intestinal mucosa, histamine functions as a central neuromodulator and immunoregulatory molecule, with well-established roles in microglial activation, blood–brain barrier permeability, and neuroinflammatory signaling [87,88]. Chronic colitis is associated with increased mast cell activation and enhanced histamine release, which, in the context of systemic inflammation and increased intestinal permeability, may contribute to elevated circulating histamine levels.

### 4.3. Musculoskeletal Disorders

Experimental studies indicate that chronic colonic inflammation may exert systemic effects on peripheral tissues, including skeletal muscle [89], using trinitrobenzene sulfonic acid (TNBS)- and dextran sodium sulfate (DSS)-induced rodent models of colitis, demonstrated that sustained intestinal inflammation induces a systemic increase in reactive oxygen species (ROS) and pro-inflammatory cytokines such as TNF-α, IL-6, and IL-1β. This inflammatory milieu disrupts redox homeostasis in peripheral tissues, including skeletal muscle, suggesting that colitis-associated oxidative stress extends beyond the intestinal compartment [90,91]. Similarly, Sui et al. [92] reported that gut microbiota aggravate TNBS-induced colitis and remote organ injury via oxidative stress mechanisms, further supporting the concept of systemic redox imbalance during chronic intestinal inflammation [93,94,95,96,97,98,99].

Alterations in total oxidative status (TOS) observed in colitis models may reflect compensatory changes in mitochondrial function and antioxidant defenses in skeletal muscle under persistent systemic inflammation [100]. Experimental data indicate that chronic inflammatory states are associated with modulation of key antioxidant enzymes, including superoxide dismutase (SOD), catalase (CAT), and glutathione S-transferase (GST), alongside reduced glutathione (GSH) levels in muscle tissue [101,102]. For instance, Wei et al. [103] reported demonstrated that elevated ROS levels activate nuclear factor erythroid 2-related factor 2 (Nrf2), leading to upregulation of antioxidant enzymes in skeletal muscle.

Although direct mechanistic evidence linking histamine, colitis, and skeletal muscle dysfunction remains limited, histamine signaling has been shown to influence skeletal muscle physiology and adaptive responses, particularly under inflammatory or stress conditions [104,105]. Collectively, these findings support the hypothesis that chronic colitis may contribute to secondary musculoskeletal dysfunction through oxidative stress-mediated mechanisms, potentially modulated by histaminergic signaling pathways.

### 4.4. Respiratory Disorders

Recent research has revealed a link between chronic colonic inflammation and respiratory dysfunction, attributed to mechanisms such as systemic inflammation, oxidative stress, and bidirectional immune signaling along the gut–lung axis [106,107]. Persistent immune activation in the colon leads to elevated systemic levels of pro-inflammatory cytokines and reactive oxygen species (ROS), which can disturb redox balance in remote organs, including the lungs [108]. Additionally, heightened intestinal permeability promotes microbial translocation and endotoxemia, further amplifying systemic inflammatory responses and modulating pulmonary immune homeostasis [109,110]. Clinically, associations have been observed between colitis and various respiratory manifestations, including airway inflammation, bronchial hyperreactivity, and interstitial lung changes [111].

Oxidative stress is a recognized driver of pulmonary inflammation and airway remodeling, and systemic ROS of intestinal origin may contribute to increased vulnerability of lung tissue [112,113,114]. Within this context, histamine emerges as a biologically plausible mediator linking colitis with respiratory pathology [115,116,117,118]. As a key signaling molecule involved in bronchoconstriction, vascular permeability, and airway inflammation—primarily through H1 and H4 receptors—histamine may exacerbate oxidative stress and immune activation in the lungs under inflammatory conditions [15,118]. While direct evidence for a causal role of colitis-associated histamine in respiratory disorders remains limited, insights from gut–lung axis research increasingly support a mechanistic interconnection between chronic intestinal inflammation and pulmonary dysfunction.

### 4.5. Cardiovascular and Thromboembolic Disorders

Chronic colonic inflammation is increasingly recognized as a contributor to cardiovascular dysfunction, reflecting the systemic reach of persistent intestinal immune activation. Sustained inflammation in the colon induces systemic TNF-α/IL-6-driven nicotinamide adenine dinucleotide phosphate (NADPH) oxidase activation, increasing circulating ROS, which promotes endothelial nitric oxide depletion, endothelial nitric oxide synthase (eNOS) uncoupling, and vascular oxidative injury [119,120,121,122,123,124]. Concurrent mast cell activation and histamine release further enhance H1-dependent endothelial permeability and leukocyte recruitment, amplifying vascular inflammation and prothrombotic signaling [124,125,126,127]. Patients with IBD may exhibit metabolic disturbances, including alterations in lipid profiles and features of metabolic syndrome, particularly in ulcerative colitis, contributing to a proatherogenic environment [128,129]. Pro-inflammatory cytokines such as TNF-α and IL-1 promote endothelial dysfunction, reduce nitric oxide bioavailability, and increase arterial stiffness, thereby elevating the risk of ischemic heart disease, stroke, and heart failure [130,131]. Additionally, IBD is associated with a heightened risk of thromboembolic events, driven by platelet activation, endothelial injury, and a procoagulant state, particularly during disease exacerbations and glucocorticosteroid treatment [132,133,134,135,136].

The breakdown of intestinal barrier function and subsequent microbial translocation further amplify systemic inflammation, directly compromising vascular homeostasis. Endothelial cells exposed to sustained inflammatory and oxidative signals undergo functional changes that favor vascular remodeling and inflammation [137,138,139]. Within this context, histamine emerges as a potential molecular link between colonic inflammation and cardiovascular pathology. As a key modulator of vascular tone and permeability, histamine—primarily via H1 and H2 receptors—regulates endothelial function, smooth muscle activity, and immune cell trafficking [124,140,141,142,143,144]. In chronic inflammatory states, enhanced histaminergic signaling may exacerbate oxidative stress and contribute to vascular dysfunction.

Taken together, these interconnected mechanisms—systemic inflammation, oxidative stress, endothelial activation, and histamine-mediated effects—suggest that chronic colitis may significantly heighten cardiovascular vulnerability.

## 5. Histamine Signaling in the Gut

Histamine is a pleiotropic biogenic amine that plays a fundamental role in gastrointestinal physiology and immune regulation [11]. Within the intestinal environment, histamine is produced primarily by mast cells, enterochromaffin-like cells, and, under certain conditions, by histamine-producing gut microbiota [12,13,14,15,16]. Beyond its classical role in gastric acid secretion, histamine participates in the regulation of epithelial barrier integrity, intestinal motility, vascular tone, and mucosal immune responses [145,146]. Histamine exerts its biological effects through four G-protein-coupled receptors (H1–H4), all of which are expressed to varying degrees in intestinal tissues [147].

Under physiological conditions, histamine signaling contributes to mucosal defense and immune surveillance [148,149]. In chronic colonic inflammation, however, histamine production becomes dysregulated and receptor expression patterns are altered. Increased mast cell density has been consistently reported in ulcerative colitis and experimental colitis [150], alongside enhanced histidine decarboxylase (HDC) activity in inflamed mucosa, indicating elevated local histamine synthesis [25]. Elevated histamine levels may amplify mucosal inflammation by promoting granulocyte recruitment and cytokine release through H1- and H4-dependent mechanisms [151,152]. Moreover, histamine can enhance oxidative stress and disrupt epithelial tight junction integrity, thereby increasing barrier permeability and facilitating microbial translocation [153,154,155,156].

Importantly, the pharmacological effects of antihistamines depend on receptor subtype selectivity, tissue distribution, and downstream signaling mechanisms [157,158]. Classical H1 and H2 receptor antagonists primarily affect pathways related to acute inflammatory responses and gastric acid secretion; however, their therapeutic efficacy in chronic colitis remains inconsistent, potentially due to limited modulation of deeper immune and oxidative pathways [147]. In contrast, H3 and H4 receptors are more closely associated with neuroimmune signaling, leukocyte recruitment, cytokine regulation, and oxidative stress modulation within inflamed tissues, suggesting that selective H3/H4-targeted therapies may provide a more mechanistically focused approach in colitis [159].

### 5.1. Histamine 1 Receptor

Functionally, H1R signaling couples classically to Gq/PLC–IP3/Ca^2+^ pathways, translating histamine exposure into smooth muscle contraction, enhanced vascular permeability, and pro-inflammatory responses [160]. In the gut wall, H1R contributes to contractile responses of intestinal smooth muscle (with evidence of coexistence of H1-mediated Ca^2+^-dependent contraction and H2-mediated cyclic adenosine monophosphate (cAMP)-dependent relaxation), providing a mechanistic basis for cramping-like motility effects during mucosal inflammation [161,162]. In parallel, H1R-linked increases in microvascular permeability and fluid/ion transport are mechanistically compatible with inflammatory diarrhea phenotypes, and earlier experimental work has discussed H1 antagonism (e.g., pyrilamine) as a way to dampen mast cell-histamine-driven secretory/transport components in inflammatory gut states [163]. Histamine can directly modulate antigen-presenting cell function; H1R signaling in dendritic cells influences their activation state and cytokine output, thereby shaping subsequent T-cell priming and effector polarization [15,163,164]. In human gut tissue, H1R has been reported among the most consistently expressed histamine receptor subtypes, with disease-associated alterations in histamine receptor expression patterns described in gastrointestinal disorders, including inflammatory bowel disease [148,165]. In the epithelial context, colon-derived epithelial models frequently show predominant H1R expression, supporting the concept that histamine can directly engage epithelial programs relevant to barrier function and inflammatory signaling [166]. Translating this to colitis, where mucosal mast cell activation and elevated histamine availability have been reported, H1R engagement on antigen-presenting cells and local vascular/epithelial targets is biologically plausible as an amplifier of inflammatory loops (permeability → immune cell recruitment → cytokine production → further barrier disruption) [59,167].

### 5.2. Histamine 2 Receptor

Histamine H2 receptors (H2R) contribute to epithelial secretion and modulation of immune cell function [168]. Signaling is primarily coupled to Gs proteins, leading to activation of adenylate cyclase and increased intracellular cAMP levels, a pathway generally associated with anti-inflammatory and barrier-stabilizing responses [169,170,171,172]. In the intestinal epithelium, H2R activation can influence ion transport and secretory processes, contributing to fluid homeostasis [173,174,175,176,177]. More importantly in the context of colitis, H2R signaling modulates immune cell function. Histamine acting through H2R has been shown to suppress excessive pro-inflammatory cytokine production in certain immune cell populations, including dendritic cells and T lymphocytes, thereby influencing T-cell polarization and immune tolerance [178,179,180,181]. In experimental colitis models, alterations in H2R expression and histamine availability have been observed, indicating that dysregulated histamine signaling may shift the balance between pro- and anti-inflammatory pathways. Some studies suggest that H2R activation may attenuate mucosal inflammation, while others indicate that receptor-specific responses depend on disease stage and cellular context. This duality highlights the complexity of histaminergic signaling in colitis and underscores the need to consider receptor-specific targeting rather than global histamine blockade [182,183]. However, these findings should be interpreted with caution, as the biological role of H2R signaling is not necessarily equivalent to the overall effects of H2 receptor antagonists observed in clinical or experimental settings. The apparent discrepancy may reflect differences in disease stage, cellular targets, receptor expression patterns, and off-target pharmacological effects. Thus, while endogenous H2R signaling appears to exert anti-inflammatory functions under certain conditions, the therapeutic consequences of H2 receptor blockade in colitis remain incompletely understood and may be highly context dependent.

### 5.3. Histamine 3 Receptor

Histamine H3 receptors (H3R) are primarily expressed on neuronal elements and function as presynaptic autoreceptors and heteroreceptors regulating neurotransmitter release [184,185,186,187]. Within the gastrointestinal tract, H3Rs are predominantly localized in the enteric nervous system, where they modulate enteric neurotransmission, intestinal motility, and neurogenic secretion [188,189]. H3R signaling is coupled to Gi/o proteins, leading to inhibition of adenylate cyclase, reduced intracellular cAMP levels, and suppression of calcium influx, thereby dampening neurotransmitter release [190,191,192,193,194].

In the context of colitis, neuroimmune interactions play a critical role in disease progression [195,196,197,198,199,200]. Mucosal inflammation is associated with altered enteric neuronal activity, visceral hypersensitivity, and dysregulated motility [201,202]. Given its inhibitory control over neurotransmitter release—including acetylcholine, substance P, and other neuropeptides—H3R may influence neurogenic inflammation and gut motor disturbances during colitis [203,204]. Experimental evidence suggests that modulation of H3R signaling can alter intestinal transit and visceral sensitivity, processes frequently disrupted in inflammatory bowel conditions [205].

Moreover, as inflammation-associated changes in the enteric nervous system contribute to sustained neurogenic signaling and cytokine production, H3R-mediated suppression of excitatory neurotransmission may exert indirect immunomodulatory effects [206,207]. Moreover, gut-specific H3R signaling has been implicated in broader neuroimmune regulatory circuits capable of modulating peripheral inflammation [208]. Findings suggest that H3R likely acts as a modulatory brake within inflammation-remodeled enteric circuits, but its net impact on mucosal inflammation is context dependent and insufficiently resolved.

### 5.4. Histamine 4 Receptor

Histamine H4 receptors (H4R) are predominantly expressed by mucosal immune cells (and, under certain conditions, also by epithelial cells) and regulate key inflammatory processes in the gut, including granulocyte recruitment and cytokine production [209]. As a Gi/o-coupled receptor, it inhibits adenylate cyclase activity, reduces intracellular cAMP levels, and promotes intracellular calcium mobilization via βγ-subunit signaling. This signaling cascade triggers cytoskeletal rearrangement and integrin activation, thereby promoting directed leukocyte migration (chemotaxis) toward histamine gradients; in colitis, this is highly pertinent because mucosal damage is driven by recruitment and activation of granulocytes and other innate effector cells [159,210]. In addition to effects on antigen-presenting cells, histamine also targets lymphocyte subsets. H4R is functionally expressed on human CD4+ T cells (including memory/effector populations) and regulates T-cell migration and effector programs; notably, H4R signaling modulates Treg chemotaxis and suppressor activity, supporting a role in immune homeostasis and surveillance [211,212]. Pharmacological H4R antagonism attenuates TNBS colitis in rats (reduced myeloperoxidase (MPO) and TNF-α), supporting a pro-inflammatory H4R axis; however, genetic and model-dependent data indicate that H4R signaling can also exert protective effects in acute neutrophilic colitis or in settings of impaired host defense, highlighting strong context dependence [59,151,213]. Notably, the same study argues that divergent outcomes likely reflect model- and phase-specific immunopathology rather than species effects per se, underscoring the need to stratify H4R biology by inflammatory endotype (acute innate-dominant versus chronic or adaptive-driven colitis).

Table 1 summarizes localization, signaling pathways, biological functions, and potential relevance in colitis of the discussed histamine receptor subtypes.

## 6. Antihistamine Drugs in Colitis

### 6.1. Preclinical Studies in Animal Models

#### 6.1.1. H1 Receptor Antagonists

Dithiaden, an H1 receptor antagonist, was investigated in a model of acetic acid-induced colitis in rats. In a 1999 study published in Physiological Research, pretreatment with locally (intrarectally) administered dithiaden reduced the degree of acute colonic inflammation. This effect was evidenced by a lower macroscopic score of mucosal damage, reduced colonic weight, and decreased myeloperoxidase activity, reflecting diminished leukocyte infiltration. Additionally, vascular permeability and gamma-glutamyltranspeptidase activity, elevated by acetic acid exposure, were reduced following dithiaden pretreatment. The results indicated that topically administered dithiaden may protect the colonic mucosa from an acute inflammatory insult by interfering with the action of histamine, a major mast cell mediator [214].

Mizolastine, a novel H1 receptor antagonist, was evaluated by Goldhill et al. [215] in a rat model of TNBS-induced colitis. Mizolastine administered orally at doses of 0.03–3.00 mg/kg significantly reduced nociception in response to rectal balloon distension (by 49% at 0.3 mg/kg), macroscopic colonic damage (by 78% at 3.0 mg/kg), histological damage (by 54% at 3.0 mg/kg), colonic tissue weight (by 69% at 3.0 mg/kg), and myeloperoxidase activity (by 66% at 3.0 mg/kg). Terfenadine, another H1 antagonist, used in comparison, tested at doses of 3–30 mg/kg under the same conditions, showed no significant effect. The authors suggested that mizolastine, in addition to its antiallergic properties, possesses anti-inflammatory effects that may not be exclusively related to H1 receptor blockade, and that it reduces afferent hypersensitivity, tissue damage, and neutrophil infiltration during colitis [215].

Desloratadine, a second-generation antihistamine, has been evaluated for its potential therapeutic effect in ulcerative colitis using a rat model of trinitrobenzenesulfonic acid (TNBS)-induced colitis. In a study by Bhat et al. [216], rats received oral desloratadine (10 mg/kg), 5-aminosalicylic acid (5-ASA, 25 mg/kg), or a combination of both drugs following colitis induction. The results demonstrated significant protective effects of desloratadine, particularly in combination with 5-ASA, as manifested by reduced disease activity score (DASR), colon weight-to-body weight ratio (CBWR), and improved colonic morphology. Furthermore, significant reductions in plasma and colon histamine levels, pro-inflammatory cytokines (IL-1β and TNF-α), and restoration of reduced glutathione (GSH) levels were observed, along with decreased malondialdehyde (MDA) content. The authors attributed desloratadine’s protective effects to its antihistaminic, anticytokine, and antioxidant properties [216]. The restoration of glutathione levels and reduction in lipid peroxidation markers observed following desloratadine treatment suggest activation of endogenous antioxidant defense mechanisms. Given the central role of Nrf2 in regulating cellular antioxidant responses, including glutathione synthesis and the expression of cytoprotective enzymes, Nrf2-related pathways may contribute to these effects. However, direct evidence linking desloratadine treatment with Nrf2 activation in experimental colitis models remains limited.

Fexofenadine, a well-known histamine H1 receptor antagonist, was investigated by Zhao et al. [217] in both dextran sulfate sodium (DSS)- and TNBS-induced murine models of inflammatory bowel disease. Orally administered fexofenadine demonstrated significant therapeutic effects, evidenced by mitigated clinical symptoms, decreased secretion of pro-inflammatory cytokines (IL-6 and IL-1β), lowered intestinal inflammation, and reduced activation of the NF-κB pathway (indicated by reduced p-p65 and p-IκBα). Intriguingly, this study employed a comparative genetic approach to dissect the mechanism of action. The protective effects of fexofenadine were completely lost in mice deficient in cytosolic phospholipase A2 (cPLA2) but were fully preserved in histamine H1 receptor-deficient mice. This in vivo comparison between wild-type, H1 receptor knockout, and cPLA2 knockout mice demonstrates that fexofenadine’s therapeutic effects in murine colitis models are mediated through cPLA2 and are independent of its canonical target, the histamine H1 receptor [217]. These findings are particularly important because cPLA2 is a key enzyme responsible for the release of arachidonic acid from membrane phospholipids, thereby initiating the synthesis of numerous pro-inflammatory eicosanoids. Consequently, inhibition of cPLA2 signaling may attenuate multiple downstream inflammatory pathways involved in intestinal injury. Moreover, the concomitant reduction in NF-κB activation and pro-inflammatory cytokine production suggests that the beneficial effects of fexofenadine extend beyond histamine receptor antagonism and involve broader modulation of intracellular inflammatory signaling networks [217]. Collectively, these observations support the concept that selected second-generation antihistamines may exert anti-inflammatory actions through non-canonical molecular targets independent of H1 receptor blockade [217].

#### 6.1.2. H2 Receptor Antagonists

Lafutidine, a novel histamine H2 receptor antagonist with gastroprotective properties, was investigated by Okayama et al. [218] in a rat model of dextran sulfate sodium DSS-induced colitis. Administered orally twice daily for 6 days, lafutidine dose-dependently reduced the severity of colonic inflammation, as evidenced by decreased ulcer area, improved colon length, and reduced myeloperoxidase (MPO) activity. Notably, these protective effects were mimicked by capsaicin administration and completely abolished by chemical ablation of capsaicin-sensitive sensory neurons, suggesting that lafutidine’s mechanism of action was dependent on sensory neuron activation rather than H2 receptor blockade. This interpretation was further supported by the finding that cimetidine, a classical H2 receptor antagonist (H2RA), failed to exert any protective effect in the same model, indicating that the therapeutic potential observed with lafutidine is not mediated through H2 receptor antagonism but rather through stimulation of sensory neurons and subsequent enhancement of mucus secretion in the colonic mucosa [218].

Cimetidine, (40 mg/day, orally for 7 days) significantly enhanced ulcer healing in the rat acetic acid-induced chronic gastric ulcer model, consistent with its antisecretory properties. Capsaicin (5 mg/day) likewise improved healing; however, combined treatment with capsaicin and cimetidine was less effective than capsaicin alone. Mechanistically, cimetidine reduced gastric mucosal blood flow in a dose-dependent manner and attenuated capsaicin-induced hyperemia, while producing dose-related suppression of acid secretion. Capsaicin did not influence acid output. These findings indicate that H2 receptor antagonism promotes mucosal repair primarily through acid suppression but may interfere with hyperemia-mediated healing mechanisms by reducing mucosal perfusion [219].

Tarras et al. [220] assessed impact on intestinal barrier function in the context of Clostridium difficile toxin exposure. Using HT29-MTX mucus-producing colonic epithelial cell monolayers, they demonstrated that pre-incubation with cimetidine (3 µM) for 48 h significantly reduced mucus content from 5.0 ± 0.5 to 1.3 ± 0.4 ng/mL (*p* < 0.001). This was associated with increased uptake of toxin A (106.6 ± 6.5 ng/mL vs. 32.8 ± 3.2 ng/mL in controls), elevated apoptosis rates (17.1 ± 1.1% vs. 8.2 ± 0.4%), and enhanced monolayer permeability (1.02 ± 0.07 nmol/cm^2^/h vs. 0.56 ± 0.02 nmol/cm^2^/h). These findings suggest that cimetidine, and potentially other H2RA, may compromise the protective mucus layer, increasing epithelial susceptibility to bacterial toxin-induced damage [220].

Famotidine, a selective H2 receptor antagonist, was evaluated in a mechanistic study of inflammatory bowel disease, demonstrating that histamine receptor 2 (H2R) signaling is required to suppress innate immune responses to bacterial ligands. In this work, blockade of H2R with famotidine impaired the regulatory effects of endogenous histamine on antigen-presenting cells and T cells. Specifically, H2R signaling was shown to limit pro-inflammatory cytokine production in response to Toll-like receptor (TLR) stimulation. Pharmacological inhibition of H2R enhanced innate immune activation, leading to increased secretion of cytokines such as IFN-γ and IL-17, and amplified mucosal inflammation in experimental models. In murine T-cell transfer colitis, loss of H2R signaling resulted in exacerbated disease severity, characterized by greater weight loss and higher histological inflammation scores. These findings indicate that, H2R exerts an immunomodulatory and potentially protective role in intestinal inflammation. Consequently, pharmacological H2 antagonism (e.g., famotidine) may remove an endogenous anti-inflammatory brake within the gut mucosa, highlighting the context-dependent effects of histaminergic signaling in colitis [182].

Kawashima et al. [221] investigated the broader class of H2 receptor antagonists in a mouse model of non-steroidal anti-inflammatory drug (NSAID)-induced enteropathy. While not a classic colitis model, this study provides important insights into how H2RA may influence intestinal homeostasis during inflammatory states. Administration of indomethacin alone increased the abundance of Erysipelotrichaceae and decreased Clostridiales in the gut microbiota, creating a dysbiotic environment conducive to inflammation. Concurrent administration of an H2RA (representative of the class, including agents such as famotidine) normalized these microbial changes, restoring the balance of bacterial populations and suggesting that H2RA may exert indirect anti-inflammatory effects through modulation of the gut microbiome [221].

#### 6.1.3. H3 Receptor Antagonists

Thioperamide, a combined H3/H4 receptor antagonist, was investigated by Fogel et al. [205] in a rat model of trinitrobenzene sulfonic acid (TNBS)-induced colitis to evaluate its effects on regional hemodynamics. In this study, presented at a conference and published in Inflammation Research, the researchers assessed the influence of thioperamide on blood flow parameters in the inflamed colon. The findings, though briefly reported in an abstract, contribute to the limited body of evidence on H3 receptor modulation in colitis, suggesting that H3/H4 receptor antagonism may influence vascular responses during intestinal inflammation [205].

M39, a novel selective histamine H3 receptor antagonist/inverse agonist, was characterized pharmacologically by Bastaki et al. [222] in a gastroprotection study using C57BL/6 mice. The study provides detailed quantitative data on M39’s pharmacological profile. The compound demonstrated a high in vitro H3 receptor antagonist affinity (hH3R pKi = 7.62), a high selectivity profile, and high in vivo H3 receptor antagonist potency with an ED50 of 2.7 ± 1.0 mg/kg following oral administration. In the experimental protocol, acute systemic administration of the H3 receptor agonist (R)-α-methylhistamine (RAMH, 100 mg/kg, i.g.) significantly reduced the severity of macroscopically assessed ulcer index, increased gastric acid output, and increased mucosal prostaglandin E2 (PGE2) production without altering somatostatin concentration in gastric juice (all *p* < 0.05). Acute systemic administration of M39 (0.3 mg/kg, i.g.) dose-dependently (0.3–3 mg/kg, i.g.) abrogated the RAMH-induced increase of acid output (*p* < 0.05) but did not affect dimaprit (H2 receptor agonist)-stimulated acid secretion, confirming that M39 modulates gastroprotective effects specifically through interactions with histamine H3 receptors. Additionally, the RAMH-induced increase in PGE2 production was reversed when mice were pretreated with M39 (0.3 mg/kg, o.p.) (*p* < 0.05), demonstrating that changes in histamine and gastric acid secretion induced by H3 receptor activation are reflected in alterations of PGE2 biosynthesis. While this study was conducted in a gastric ulcer model rather than colitis, the findings establish that H3 receptors are profoundly involved in the maintenance of gastrointestinal mucosal integrity by modulating PGE2 and gastric acid secretion. The demonstration that M39 can modulate PGE2 production—a critical mediator in intestinal inflammation—suggests potential relevance for future investigations in colitis models [222].

In contrast to other histamine receptor subtypes, there is currently no robust preclinical evidence demonstrating the therapeutic efficacy of selective H3 receptor antagonists in established animal models of colitis. The available data are limited to a small number of studies using mixed H3/H4 ligands (e.g., thioperamide), primarily examining hemodynamic or neurogenic parameters rather than canonical inflammatory endpoints such as cytokine production, histological scores, or disease activity indices. Consequently, a clear translational framework for targeting H3 receptors in intestinal inflammation remains undeveloped, highlighting a significant gap between mechanistic neuroimmune hypotheses and pharmacological validation in colitis models.

#### 6.1.4. H4 Receptor Antagonists

JNJ 10191584 (VUF6002), a potent and highly selective histamine H4 receptor antagonist with 540-fold selectivity over the H3 receptor (binding Ki of 26 nM), was investigated by Varga et al. [151] in a rat model of trinitrobenzene sulfonic acid (TNBS)-induced colitis. Administered orally twice daily at doses ranging from 10 to 100 mg/kg, JNJ 10191584 caused a dose-dependent reduction in macroscopic damage of the colonic tissue. The treatment significantly inhibited the TNBS-provoked elevation of both colonic myeloperoxidase (MPO) activity (a marker of neutrophil infiltration) and tumor necrosis factor-alpha (TNF-α) levels. Histological assessment further revealed a reduction in the increase in mucosal and submucosal thickness, as well as in neutrophil infiltration, in animals treated with the antagonist [151].

JNJ 7777120, another well-characterized, highly selective histamine H4 receptor antagonist, was evaluated in the same study by Varga et al. [151] using the TNBS-induced colitis model in rats. When administered orally at 100 mg/kg twice daily, JNJ 7777120 significantly reduced macroscopic colonic injury. This protective effect was accompanied by a marked inhibition of the increased colonic myeloperoxidase (MPO) activity and TNF-α levels provoked by TNBS instillation. The findings from both JNJ 10191584 and JNJ 7777120 experiments indicated a pro-inflammatory role for the histamine H4 receptor in this colitis model, suggesting that its antagonism represents a novel pharmacological approach for treating colitis [151].

LINS01007, a novel H4 receptor antagonist (pKi 6.2), was investigated by Lippi et al. [223] in a mouse model of dextran sulfate sodium (DSS)-induced ulcerative colitis. The study involved male BALB/c mice with acute colitis induced by 3% DSS in drinking water for six days, while the test compound was administered daily via intraperitoneal injection at a dose of 5 mg/kg. Animals treated with LINS01007 showed a prevention of the reduction in water and food intake observed in control mice from day four (*p* < 0.05). Histological analysis revealed that signs of edema, hyperplasia, disorganized intestinal crypts, and neutrophilic infiltrations were significantly reduced in treated animals. Furthermore, significant reductions were observed in the serum and tissue levels of key inflammatory markers: prostaglandin E2 (PGE2), cyclooxygenase-2 (COX-2), interleukin-6 (IL-6), nuclear factor kappa-B (NF-κB), and signal transducer and activator of transcription 3 (STAT3). The results demonstrated significant protective effects of LINS01007 against DSS-induced colitis, highlighting the potential of H4 receptor antagonism as a promising treatment approach for ulcerative colitis [223].

Contrasting evidence from genetic studies provides additional context for understanding H4 receptor function in colitis. Wunschel et al. [213] investigated the impact of H4 receptor deficiency using H4 receptor knockout mice in a TNBS-induced acute colitis model. Contrary to the pharmacological studies with antagonists, this genetic approach revealed that the lack of H4 receptor expression aggravated acute colitis symptoms in mice. H4-deficient animals exhibited more severe disease manifestations compared to wild-type controls, suggesting that under certain conditions or during specific phases of the inflammatory response, H4 receptor signaling may exert anti-inflammatory or protective effects. This discrepancy between pharmacological antagonism and genetic deletion underscores the complexity of H4 receptor biology and highlights the need for further studies to clarify the receptor’s precise role at different stages and in different models of intestinal inflammation. The contrasting findings may reflect fundamental differences between transient pharmacological receptor blockade and lifelong receptor deficiency, which may induce compensatory adaptations affecting immune homeostasis [213]. Furthermore, H4 receptors are expressed on multiple immune cell populations, including mast cells, eosinophils, dendritic cells, neutrophils, and T lymphocytes, where they regulate both pro-inflammatory and immunoregulatory responses. Consequently, H4 receptor signaling may simultaneously promote leukocyte recruitment and tissue inflammation while also contributing to immune regulation and host defense. The overall effect of H4 receptor modulation is therefore likely determined by disease stage, inflammatory endotype, cellular context, and the balance between innate and adaptive immune responses. These observations suggest that H4 receptor signaling cannot be classified as exclusively pro- or anti-inflammatory and may instead function as a context-dependent regulator of intestinal immune responses. Future therapeutic strategies may therefore require selective or temporally targeted modulation of H4 receptor activity rather than complete and sustained receptor inhibition.

#### 6.1.5. Comprehensive Studies

JNJ7777120 and levocetirizine, an H4 receptor antagonist and an H1 receptor antagonist, respectively, were investigated by Deiteren et al. [224] in a rat model of post-inflammatory visceral hypersensitivity following trinitrobenzene sulfonic acid (TNBS)-induced colitis. The experimental protocol involved male Sprague–Dawley rats with colitis induced by intrarectal administration of 30 mg TNBS. Only animals showing endoscopic healing by day 14 were included in the post-inflammatory group, ensuring that observed hypersensitivity was truly “post-inflammatory” rather than reflecting ongoing active inflammation. Visceromotor responses (VMRs) to colorectal distension (CRD) at pressures of 10–60 mmHg were evaluated as a measure of visceral pain perception, with EMG electrodes implanted in the abdominal musculature [224].

JNJ7777120, administered intraperitoneally 30 min prior to VMR assessment at doses of 1, 10, and 30 mg/kg, produced a dose-dependent reduction in the enhanced VMRs to CRD in post-colitis rats. At 30 mg/kg, the compound completely normalized VMRs to levels comparable to control rats, with significant effects observed at distension pressures of 20 mmHg (*p* < 0.01), 40 mmHg (*p* < 0.001), and 60 mmHg (*p* < 0.001). The 10 mg/kg dose produced intermediate effects, while 1 mg/kg was ineffective, demonstrating that H4 receptor activation is necessary for the maintenance of post-inflammatory visceral hypersensitivity [224].

Levocetirizine, administered intraperitoneally at doses of 0.1, 0.3, and 1 mg/kg, likewise produced a dose-dependent reduction in the enhanced VMRs. The highest dose (1 mg/kg) significantly reduced VMRs at 20 mmHg (*p* < 0.05), 40 mmHg (*p* < 0.01), and 60 mmHg (*p* < 0.01), confirming that H1 receptor signaling also contributes to visceral pain sensitization following colitis. The 0.3 mg/kg dose showed partial effects, while 0.1 mg/kg was ineffective [224].

Combined administration of both compounds at subtherapeutic doses (JNJ7777120 at 10 mg/kg and levocetirizine at 0.3 mg/kg) produced a significant reduction in VMRs that was greater than the sum of individual effects, indicating synergistic interaction between H4 and H1 receptor blockade. At distension pressures of 40 and 60 mmHg, the combination reduced VMRs by approximately 60–70% (*p* < 0.01 vs. vehicle), achieving statistical significance where neither drug alone was effective [224].

Mechanistic investigations revealed that post-colitis rats displayed a significantly higher number of colonic mast cells (25.3 ± 2.1 vs. 14.7 ± 1.8 cells/mm^2^ in controls, *p* < 0.01) and excessive histamine release (2.8 ± 0.3 vs. 1.2 ± 0.2 ng/mg tissue, *p* < 0.01). Treatment with JNJ7777120 at 30 mg/kg significantly reduced histamine release to 1.6 ± 0.2 ng/mg tissue (*p* < 0.05). Gene expression analysis by quantitative RT-PCR showed that in the colon, both H4R and H1R mRNA were present, while in the dorsal root ganglia, only H1R mRNA was found. Importantly, colonic H4R mRNA expression was significantly upregulated in post-colitis rats (2.3 ± 0.4-fold increase, *p* < 0.05), suggesting increased importance of this receptor during post-inflammatory states [224].

The authors concluded that H4R and H1R antagonists dose-dependently reduce and even normalize post-inflammatory visceral hypersensitivity via different underlying mechanisms, but with a synergistic effect. The upregulation of colonic H4R expression and the absence of H4R from sensory neurons indicate a peripheral site of action within the colon, while H1R effects may involve both peripheral and central sites. Both receptor subtypes represent promising targets for the treatment of post-inflammatory visceral hypersensitivity, and combination therapy may offer therapeutic advantages [224].

The study has important limitations: it assessed visceral hypersensitivity at only a single time point (day 14) using acute drug administration rather than chronic dosing, included only male rats (limiting generalizability to female populations), lacked pharmacokinetic data to guide translation to human dosing, tested only one compound per receptor class, and did not include histological assessment of colonic tissue following drug treatment. Despite these limitations, the rigorous experimental design and integration of functional, cellular, and molecular endpoints make this study the most methodologically advanced comparative investigation of H4 and H1 receptor antagonists in post-inflammatory visceral hypersensitivity published to date [224].

Bogielski et al. [225] conducted a comprehensive investigation examining the impact of H1 (cetirizine), H2 (ranitidine), H3 (iodophenpropit), or H4 (JNJ7777120) receptor antagonists on oxidative stress markers in liver and muscle tissue using a rat model of trinitrobenzene sulfonic acid (TNBS)-induced colitis. The study provides the most complete comparative analysis to date of how different histamine receptor subtypes modulate systemic oxidative stress during colonic inflammation. The experimental protocol involved 60 adult male Wistar rats divided into control and colitis experimental groups, with animals receiving intramuscular injections of specific antagonists targeting each histamine receptor subtype following colitis induction. The comprehensive design allowed for direct comparison of receptor-specific effects on oxidative stress parameters in peripheral tissues, offering insights into the systemic consequences of histamine receptor modulation beyond the local intestinal environment [225].

H1 receptor antagonist effects were evaluated through analysis of skeletal muscle and liver tissue samples. In skeletal muscle of control rats, H1 receptor antagonism significantly increased the activities of key antioxidant enzymes: superoxide dismutase (SOD) increased by approximately 35% and catalase (CAT) increased by approximately 28% compared to untreated controls. Parameters related to glutathione metabolism were also enhanced, including elevated levels of reduced glutathione (GSH) and increased activity of glutathione S-transferase (GST), indicating that H1 receptor blockade enhances baseline antioxidant defenses in muscle tissue. In rats with chemically induced colitis, H1 receptor antagonists specifically elevated CAT activity in skeletal muscle, suggesting a targeted enhancement of hydrogen peroxide-scavenging capacity during inflammation. In the liver, however, H1 receptor antagonists attenuated the colitis-induced hyperactivity of SOD but paradoxically depleted GSH levels, demonstrating tissue-specific and context-dependent effects on the antioxidant system [225].

H2 receptor antagonist effects demonstrated the most pronounced hepatoprotective profile among all tested compounds. In the liver tissue of colitis-induced rats, H2 receptor antagonism significantly reduced oxidative damage by decreasing malondialdehyde (MDA) levels by approximately 40% compared to untreated colitis controls. Among all four receptor subtype antagonists, the H2 receptor antagonist most effectively mitigated hepatic oxidative injury, highlighting its potential as a therapeutic target for protecting against colitis-associated systemic oxidative stress. Analysis of skeletal muscle samples revealed that H2 receptor antagonists, similar to H1 antagonists, increased SOD and CAT activities as well as glutathione metabolism parameters in control rats, indicating broad antioxidant enzyme induction independent of inflammatory conditions [225].

H3 receptor antagonist effects revealed a distinctive pattern of redox modulation. In the liver tissue of colitis-induced rats, H3 receptor antagonism produced a unique dual effect: it significantly increased GSH levels (indicating enhanced antioxidant capacity) while simultaneously elevating MDA concentrations (a marker of lipid peroxidation). This paradoxical finding suggests that H3 receptor blockade may activate competing redox pathways, enhancing glutathione-dependent antioxidant defenses while failing to prevent or even contributing to membrane lipid damage. The complex pattern observed with H3 antagonism differs markedly from the more straightforward protective effects seen with H2 antagonists and the tissue-specific effects of H1 blockade, reflecting the unique neuronal localization and presynaptic regulatory functions of H3 receptors compared to other histamine receptor subtypes [225].

H4 receptor antagonist effects in the comprehensive study demonstrated yet another distinct redox profile. Administration of the H4 receptor antagonist to colitis-induced rats resulted in increased reduced glutathione (GSH) levels in liver tissue, indicating enhanced antioxidant capacity through the glutathione system. However, this was accompanied by elevated MDA levels, similar to the pattern observed with H3 antagonism. This finding suggests that H4 receptor blockade, like H3 antagonism, may activate competing redox pathways that enhance certain aspects of antioxidant defense while leaving the liver vulnerable to lipid peroxidation. The parallel effects of H3 and H4 antagonists in this study are particularly interesting given that these two receptor subtypes are structurally related and were historically targeted by non-selective compounds such as thioperamide, though they have distinct tissue distributions and physiological functions [225].

Comparative analysis across receptor subtypes revealed that each histamine receptor antagonist produced a unique fingerprint of redox changes in peripheral tissues during colitis. H1 antagonists showed tissue-specific effects with muscle CAT elevation and hepatic SOD attenuation. H2 antagonists demonstrated the most consistent hepatoprotective profile with significant MDA reduction. H3 and H4 antagonists both paradoxically increased GSH while elevating MDA, suggesting similar but not identical effects on hepatic redox balance. These findings demonstrate that histamine receptor antagonists modulate oxidative stress responses in a receptor-dependent and tissue-specific manner, with H2 antagonists showing the greatest potential for mitigating colitis-associated systemic oxidative injury. The study underscores the complexity of histamine receptor signaling in inflammatory conditions and suggests that therapeutic strategies targeting histamine in colitis must consider not only local intestinal effects but also systemic consequences on oxidative stress in peripheral organs such as the liver and skeletal muscle.

Methodological strengths and limitations of this comprehensive study include the use of a well-established TNBS-induced colitis model, simultaneous evaluation of all four histamine receptor subtypes under identical experimental conditions, and assessment of multiple oxidative stress parameters providing an integrated view of redox status. However, the study did not assess direct effects on colonic inflammation itself, focusing instead on systemic oxidative stress markers in peripheral tissues [225].

### 6.2. Clinical Studies in Humans

#### 6.2.1. H1 Receptor Antagonists

Loratadine was investigated by Raithel et al. [226] as an adjunctive H1-receptor antagonist therapy in patients with active inflammatory bowel disease (IBD), with particular focus on its effects on systemic plasma histamine levels. In this randomized controlled short communication, patients with active ulcerative colitis or Crohn’s disease received standard anti-inflammatory treatment with or without additional loratadine. Plasma histamine concentrations were measured to assess whether pharmacological H1-receptor blockade would reduce circulating histamine levels and potentially modulate inflammatory activity. The authors observed that adjunctive loratadine did not result in a significant or consistent reduction in plasma histamine concentrations compared with standard therapy alone. Moreover, most measured histamine values remained within the normal range, regardless of H1-antagonist treatment. Based on these findings, the study concluded that low-dose loratadine does not meaningfully influence systemic histamine levels in active IBD and that plasma histamine may not adequately reflect local mucosal histamine signaling within the intestinal microenvironment. These results suggest that systemic H1-receptor blockade alone may be insufficient to modify disease activity and highlight the complexity of histamine-mediated pathways in intestinal inflammation [226].

Ketotifen was investigated by Jones et al. [227] in an open-label pilot study evaluating its efficacy in children with active ulcerative colitis. This prospective trial enrolled ten pediatric patients with newly or previously diagnosed mild-to-moderate ulcerative colitis, who received oral ketotifen at a dosage of 4 mg daily for eight weeks. Efficacy was assessed using a physician-determined disease severity index, as well as endoscopic and histologic examinations before and after treatment. The results demonstrated limited therapeutic benefit: symptoms improved in four patients and resolved completely in one patient, while endoscopic improvement was observed in three patients and histologic improvement in only one. Notably, five patients (50%) withdrew from the study due to lack of symptomatic improvement. Interestingly, increased eosinophil counts on baseline rectal biopsy were present in two of the five responders, suggesting that patients with an eosinophilic component to their inflammation may be more likely to benefit from ketotifen therapy. No adverse events were identified throughout the study period. The authors concluded that low-dose ketotifen offers a limited therapeutic advantage in active ulcerative colitis, with potential for enhanced efficacy in the subgroup of patients with elevated colonic mucosal eosinophil counts. They emphasized that further studies with increased dosages of this mast cell stabilizer for both acute and maintenance therapy are warranted. This pilot study remains the only published clinical trial investigating mast cell-targeted therapy in pediatric ulcerative colitis, though its open-label design, small sample size, and high withdrawal rate limit the generalizability of its findings [227]. Another research group, Marshall and Irvine [228], reported the clinical use of ketotifen—an agent with H1-receptor antagonism and mast-cell stabilizing properties—in patients with active colitis who had intolerance or allergy to 5-aminosalicylates (5-ASA) and therefore could not receive standard therapy. In this case series of three patients (one each with ulcerative colitis, Crohn’s disease, and collagenous colitis), ketotifen administration was associated with clinical improvement and remission of inflammatory symptoms despite prior 5-ASA intolerance, suggesting that mast-cell stabilization and/or histaminergic blockade may modulate disease activity in selected individuals. However, given the anecdotal nature of the evidence and the very small sample size, the authors emphasized the need for more rigorous, controlled clinical studies to clarify ketotifen’s efficacy and mechanisms of action in human inflammatory bowel disease [228].

Cetirizine (Zyrtec) was examined in a post-marketing surveillance study using FDA data that analyzed adverse event reports in men aged 50–59. Among 2811 men reporting side effects while taking cetirizine, 20 individuals (0.71%) experienced Crohn’s disease. However, this data is significantly complicated by concomitant medication use, with 80% of these patients also receiving Humira (adalimumab), a biologic therapy specifically indicated for inflammatory bowel disease. This high rate of concurrent IBD treatment strongly suggests that cetirizine use in these patients was coincidental rather than causal, occurring in individuals already diagnosed with or predisposed to IBD rather than representing drug-induced disease. The finding highlights the limitations of post-marketing surveillance data for establishing causal relationships, particularly when signal detection is confounded by indication bias and polypharmacy in patient populations with pre-existing conditions [229].

A phase 2 clinical trial is currently registered (estimated start February 2026) at Tanta University, Egypt, investigating the efficacy and safety of desloratadine as adjuvant therapy in patients with mild to moderate ulcerative colitis. The study aims to enroll 44 patients aged 18–65 with active mild to moderate UC according to American College of Gastroenterology guidelines. Participants will be randomized into two groups: a control group receiving mesalamine 1000 mg three times daily for 3 months, and a desloratadine group receiving mesalamine 1000 mg three times daily plus desloratadine 5 mg once daily for 3 months. The primary outcome measure is change in disease activity and severity assessed by the Partial Mayo Scoring Index (PMSI) and Truelove and Witt’s classification. Secondary outcomes include changes in serum biomarkers of inflammation and oxidative stress (TNF-α and MDA). The study is based on preclinical evidence demonstrating desloratadine’s anti-inflammatory and antioxidant properties in experimental ulcerative colitis, attributed to regulation of mast cell activity and inhibition of histamine release [230].

#### 6.2.2. H2 Receptor Antagonists

A groundbreaking systematic review and meta-analysis by D’sa et al. [231] examined the association between histamine-2 receptor antagonist (H2RA) use and risk of developing inflammatory bowel diseases. The analysis included 4 observational studies with a total of 8939 participants and assessed the risk of developing Crohn’s disease, ulcerative colitis, and microscopic colitis in individuals using H2RAs compared to non-users. The results demonstrated a significantly higher risk of IBD among H2RA users (OR: 2.27; 95% CI: 1.70–3.02; *p* < 0.0001). Similar associations were observed in subgroup analyses for both adults (*p* < 0.0001) and children (*p* = 0.04). The quality of included studies was rated as “fair to good.” The authors concluded that there is a significant association between H2RA use and increased risk of developing IBD, emphasizing the need for further observational studies with large populations to confirm these findings In the analyzed meta-analysis, the authors did not undertake an in-depth mechanistic discussion explaining why H2 receptor antagonists (H2RAs) might be associated with an increased risk of inflammatory bowel disease (IBD). This is likely attributable to the epidemiological and observational nature of the included studies, which were designed to assess statistical associations rather than to investigate underlying biological mechanisms. Consequently, causal pathways were not experimentally evaluated within the scope of the review [231].

Nevertheless, several biologically plausible mechanisms may explain this association. First, H2RAs suppress gastric acid secretion, leading to increased intragastric pH and potentially altering the composition of the intestinal microbiota. Such changes may promote bacterial overgrowth, impair colonization resistance, and increase mucosal immune stimulation, all of which have been implicated in IBD pathogenesis [232,233]. Second, histamine signaling through the H2 receptor is known to exert immunomodulatory effects, generally promoting cyclic AMP (cAMP)-mediated anti-inflammatory signaling in immune cells. Pharmacological blockade of H2 receptors may therefore attenuate these regulatory pathways, potentially enhancing pro-inflammatory cytokine production and shifting immune responses toward a Th1/Th17-dominant profile [234,235]. Finally, disruption of histamine-mediated regulation of epithelial barrier function may contribute to altered intestinal permeability, thereby facilitating mucosal immune activation [57,236,237,238].

Another study also investigated the relationship between H2 receptor antagonists (H2RAs) and colitis, although from a different clinical perspective [239]. In a large pooled individual patient-level analysis of ten randomized controlled trials in moderate-to-severe ulcerative colitis, the authors examined whether baseline use of concomitant medications—including H2Ras—modifies the efficacy or safety of biologic and small-molecule therapies. In contrast to observational data suggesting an association between H2RA use and increased IBD risk, this trial-based analysis did not demonstrate a significant impact of H2RA exposure on clinical remission rates or adverse outcomes. Importantly, this study did not address disease induction but rather treatment response in patients with established ulcerative colitis. Therefore, while epidemiological data raise the possibility that H2 receptor blockade may be linked to increased IBD risk, randomized clinical trial data suggest that concomitant H2RA use does not meaningfully alter disease course or therapeutic outcomes in diagnosed UC. These findings highlight the distinction between potential effects on disease initiation and modulation of established inflammatory activity.

Another study also stands in contrast to the previously cited meta-analysis. Zylberberg et al. conducted a multicentre retrospective case–control study evaluating the association between medication use and the risk of microscopic colitis (MC) [240]. This study, published in Alimentary Pharmacology & Therapeutics, identified patients who underwent colonoscopy over a 10-year period at two academic medical centers (Columbia University Medical Center and Mayo Clinic). Cases included 344 patients with biopsy-proven microscopic colitis, matched by age, gender, and calendar period to 668 controls who underwent colonoscopy for evaluation of diarrhea but had biopsies negative for MC. After adjusting for smoking, the analysis revealed a statistically significant inverse association between H2 blocker use and microscopic colitis (OR 0.46; 95% CI 0.24–0.88). This finding indicates that patients using H2 receptor antagonists were approximately 54% less likely to have microscopic colitis compared to controls. The study also found inverse associations with proton pump inhibitors (PPIs) (OR 0.64; 95% CI 0.47–0.87) and oral diabetes medications (OR 0.47; 95% CI 0.27–0.81), while nonsteroidal anti-inflammatory drug (NSAID) use was positively associated with MC (OR 1.63; 95% CI 1.12–2.38). The authors cautiously interpreted these findings, noting that their use of a control group with diarrhea (rather than healthy controls) may have contributed to these inverse associations, and emphasized that future studies of drug-induced microscopic colitis should include control groups with diarrhea, not only healthy controls. This study is particularly noteworthy as it contradicts the commonly held assumption that H2 receptor antagonists might increase colitis risk, instead suggesting a potential protective effect, though the authors themselves urge caution in interpretation due to possible selection bias.

#### 6.2.3. H3 and H4 Receptor Antagonists

At present, there are no available publications in major medical databases describing the clinical use of selective H3 or H4 receptor antagonists in the treatment of colitis or inflammatory bowel disease (IBD) in humans. While experimental studies in animal models of colitis have suggested that pharmacological blockade of H3 and particularly H4 receptors may attenuate intestinal inflammation, these findings have not been translated into human clinical trials. Current human data are limited to studies evaluating receptor expression patterns or mechanistic insights at the tissue level, rather than interventional therapeutic applications. Therefore, despite promising preclinical evidence, the potential role of H3 and H4 receptor antagonists as therapeutic agents in human colitis remains untested and represents an important gap in translational research.

## 7. Limitations and Knowledge Gaps

Despite growing recognition of histamine’s role in intestinal inflammation and promising preclinical data, several significant limitations and knowledge gaps hinder the clinical translation of antihistamine therapy in colitis. First and most striking is the scarcity of human studies—while numerous preclinical investigations have demonstrated efficacy of H1, H2, H3, and H4 receptor modulators in animal models of colitis, clinical data remain extremely limited. The only published therapeutic trial is a small pediatric pilot study of ketotifen from 1998 [227], involving just ten patients and showing only modest benefit with a 50% withdrawal rate. A phase 2 trial of desloratadine is registered but has not yet begun recruitment. This represents a fundamental gap in translating decades of mechanistic insights into evidence-based clinical practice.

Second, available epidemiological data are conflicting and difficult to interpret. Meta-analyses suggest that H2 receptor antagonist use may be associated with increased risk of developing IBD (OR 2.27), yet a large retrospective cohort study found H2 blockers to be inversely associated with microscopic colitis (OR 0.46). These discrepancies may reflect differences in study populations, definitions of colitis, confounding factors, or true biological differences between IBD and microscopic colitis. The lack of prospective, controlled studies with clearly defined exposure and outcome measures leaves this question unresolved.

Third, the clinical potential of H3 and H4 receptor modulators remains completely unexplored in humans. Despite robust preclinical evidence—including dose-dependent effects of H4 antagonists in multiple colitis models, upregulation of H4 receptor expression in inflamed colonic tissue, and synergistic effects with H1 antagonists—no clinical studies have been conducted or registered. This gap is particularly striking given that H4 receptors are expressed in human gut immune cells and their expression is altered in inflammatory conditions.

Fourth, existing studies suffer from methodological limitations that constrain their interpretability. Preclinical studies often use acute dosing regimens that do not reflect chronic clinical use, focus primarily on male animals, and rarely include pharmacokinetic data to guide dose translation. Human data, where available, come from open-label designs, small samples, post-marketing surveillance with confounding by indication, or retrospective analyses with potential selection bias. No randomized controlled trials of antihistamines as primary or adjunctive therapy in colitis have been completed. In addition, the present article did not include a formal, tool-based quality assessment of each individual study. Given the marked heterogeneity of the available evidence (spanning diverse animal models, small pilot trials, observational cohorts, and pharmacoepidemiologic analyses) authors did not apply standardized frameworks such as the SYRCLE animal research quality scale, the Newcastle-Ottawa Scale for cohort studies, or randomized controlled trial risk-of-bias tools. As a result, the credibility of the evidence cannot be quantified in a unified manner across study types, and the conclusions of this narrative synthesis should be regarded as hypothesis generating rather than definitive.

Fifth, the optimal timing, dosing, and patient selection for antihistamine therapy remain undefined. The ketotifen pilot study suggested that patients with elevated mucosal eosinophils might respond better, but this has never been prospectively validated. Whether antihistamines would be more effective in acute flare prevention, maintenance of remission, or treatment of specific symptoms (such as visceral pain or diarrhea) is unknown. The possibility that different histamine receptor subtypes play distinct roles in different phases of inflammation—for example, H4 receptors may be more important in chronic versus acute settings—remains unexplored in clinical contexts.

Sixth, potential safety concerns have not been adequately addressed. While antihistamines are generally considered safe based on their use in allergic conditions, their long-term safety profile in IBD patients—who may have altered drug absorption, concomitant medications, and chronic inflammation affecting multiple organ systems—has not been systematically evaluated. The paradoxical finding that H2 blockers may increase IBD risk in some studies, while being protective in others, underscores the need for careful safety monitoring in future trials.

Seventh, the interaction between antihistamines and standard IBD therapies remains poorly characterized. The pooled analysis by Ahuja et al. [239] suggesting no impact on biologic efficacy is reassuring, but data for interactions with immunomodulators, corticosteroids, or 5-ASA compounds are lacking. Whether antihistamines might have additive or synergistic effects with existing therapies—as suggested by the preclinical combination of H4 and H1 antagonists—remains to be tested clinically.

Finally, the heterogeneity of colitis itself presents a challenge. Ulcerative colitis, Crohn’s disease, and microscopic colitis have distinct pathophysiologies, and histamine may play different roles in each. The upregulation of H4 receptors in inflamed colonic tissue may not be uniform across all patients or disease subtypes. Future studies will need to consider patient stratification based on biomarkers such as mast cell density, eosinophil counts, or histamine receptor expression profiles to identify those most likely to benefit.

In summary, while the preclinical rationale for antihistamine therapy in colitis is strong, the field is characterized by a striking translational gap between bench and bedside. Addressing these knowledge gaps will require well-designed, adequately powered randomized controlled trials that account for disease heterogeneity, employ rational dosing strategies based on pharmacokinetic data, and incorporate biomarker-based patient selection. Histamine-dependent signaling represents a biologically plausible and pharmacologically actionable target in colitis; however, its precise clinical relevance and therapeutic utility still require further validation in well-designed human studies. Until such studies are completed, the role of antihistamines in the management of colitis remains speculative, and their use cannot be recommended outside of clinical trials. This is further underscored by the absence of formal, standardized risk-of-bias grading in the present review, which reinforces the need for cautious interpretation of the summarized evidence.

## 8. Future Directions

Future research should aim to clarify the receptor-specific roles of histamine signaling in intestinal inflammation, with particular emphasis on translational applicability in human disease. While preclinical studies suggest that selective targeting of H3 and especially H4 receptors may attenuate experimental colitis, these findings have not yet been evaluated in well-designed human clinical trials. Prospective interventional studies assessing selective H3 and H4 receptor modulators in patients with ulcerative colitis or Crohn’s disease are therefore warranted.

In addition, future investigations should move beyond systemic measurements of circulating histamine and focus on tissue-level dynamics, including receptor expression patterns, intracellular signaling pathways, and spatial localization within the intestinal microenvironment. Single-cell transcriptomic and proteomic approaches may help delineate cell-type-specific receptor activity across epithelial, immune, and neuronal compartments. Given the emerging evidence linking histamine signaling to microbiota composition and barrier integrity, integrative studies combining immunophenotyping, microbiome profiling, and functional barrier assays will be essential.

Furthermore, longitudinal cohort studies are needed to distinguish whether histamine receptor modulation contributes primarily to disease initiation, progression, or therapeutic response. Stratified analyses based on disease phenotype, inflammatory profile (e.g., Th1/Th17 dominance), and treatment exposure may reveal context-dependent effects of specific receptor subtypes. Ultimately, a receptor-selective, precision-based approach to histamine modulation could represent a novel adjunctive strategy in inflammatory bowel disease management, provided that mechanistic clarity and clinical safety are rigorously established.

Future research should not only focus on the direct anti-inflammatory effects of histamine receptor antagonists within the intestinal mucosa, but also consider their potential impact on systemic redox homeostasis. Histamine signaling has been implicated in the modulation of oxidative pathways, including reactive oxygen species (ROS) generation and downstream inflammatory amplification. Importantly, several antihistaminic agents have demonstrated the capacity to influence cellular redox potential, either directly or indirectly through receptor-mediated immunomodulatory mechanisms.

Therefore, it is conceivable that antihistamine therapy may not necessarily produce a marked improvement in primary intestinal inflammatory activity, yet may attenuate oxidative stress-related systemic consequences of colitis. Given that colitis is increasingly recognized as a multi-organ condition characterized by oxidative imbalance, endothelial dysfunction, and extra-intestinal manifestations, future studies should evaluate whether modulation of histamine signaling contributes to the prevention of systemic tissue injury rather than solely mucosal healing.

Prospective investigations should include assessment of oxidative stress biomarkers, systemic inflammatory mediators, and organ-specific functional parameters in addition to conventional clinical remission endpoints. Such an approach may reveal therapeutic benefits that remain undetected when outcomes are restricted to intestinal disease activity scores alone. A broader redox-centered perspective could therefore redefine the potential role of antihistaminic agents as adjunctive modulators of systemic complications in colitis.

## 9. Conclusions

Colitis is a multifactorial inflammatory disorder with intestinal and systemic consequences, in which oxidative stress and immune dysregulation play central roles. This review identifies histamine as a biologically relevant mediator, acting through receptor-specific mechanisms that influence immune activation, epithelial barrier integrity, and redox balance.

Observational human data regarding H1 and H2 receptor antagonists remain inconsistent and limited in their ability to establish causality. However, experimental studies provide mechanistic evidence that modulation of histamine signaling can attenuate inflammatory responses in colitis models.

Despite robust experimental evidence—particularly concerning H3 and H4 receptors—no clinical trials to date have evaluated selective H3 or H4 antagonists in patients with colitis. In contrast, the efficacy of these compounds in animal models of intestinal inflammation is well-documented. Furthermore, existing clinical research has largely focused on intestinal disease activity as the primary endpoint, without adequately addressing the potential systemic and redox-modulating effects of antihistaminic agents.

The available evidence suggests that histamine receptor modulation may represent a multifaceted therapeutic avenue, but antihistaminic agents may not uniformly suppress primary intestinal inflammation. Their therapeutic value might lie in mitigating the oxidative stress-related systemic complications that contribute significantly to morbidity in colitis patients as documented in preclinical studies on the receptor-specific and tissue-specific effects on redox balance.

Until well-designed clinical trials incorporating both intestinal and systemic outcomes are completed, the role of antihistamines in colitis management remains speculative. Decades of mechanistic insight ought to be translated into evidence-based therapeutic strategies to offer safe, well-tolerated, and inexpensive adjunctive options for patients suffering from this chronic debilitating condition.

## Figures and Tables

**Table 1:** Histamine receptor subtypes (H1R, H2R, H3R and H4R): localization, signaling pathways, biological functions, and potential relevance in colitis [15,59,148,151,159,160,161,162,163,164,165,166,167,168,169,170,171,172,173,174,175,176,177,178,179,180,181,182,183,184,185,186,187,188,189,190,191,192,193,194,195,196,197,198,199,200,201,202,203,204,205,206,207,208,209,210,211,212,213].

Receptor	Main Localization in the Gut	Principal Signaling Pathway	Main Biological Functions	Potential Relevance in Colitis
H1R	Intestinal smooth muscle, vascular endothelium, epithelial cells, dendritic cells	Gq/PLC-IP3/Ca^2+^	Smooth muscle contraction, increased vascular permeability, regulation of epithelial and immune responses, cytokine production	May amplify inflammatory loops through increased permeability, immune cell recruitment, cytokine release, and barrier disruption
H2R	Intestinal epithelium, dendritic cells, T lymphocytes and other immune cells	Gs/adenylate cyclase/cAMP	Regulation of epithelial secretion and ion transport, modulation of immune tolerance, suppression of excessive cytokine production	May exert anti-inflammatory and barrier-stabilizing effects; however, responses appear context- and disease stage dependent
H3R	Enteric nervous system, neuronal elements	Gi/o-mediated inhibition of adenylate cyclase and calcium influx	Regulation of neurotransmitter release, intestinal motility, visceral sensitivity, and neurogenic secretion	May modulate neuroimmune interactions, visceral hypersensitivity, and inflammation-associated motility disturbances
H4R	Mast cells, granulocytes, dendritic cells, T lymphocytes, mucosal immune cells	Gi/o-mediated inhibition of adenylate cyclase with βγ-subunit signaling and calcium mobilization	Chemotaxis, leukocyte recruitment, cytokine production, regulation of T-cell migration and immune homeostasis	Strongly involved in inflammatory cell recruitment and cytokine-driven inflammation; promising therapeutic target, although effects appear context dependent

## Data Availability

No new data were created or analyzed in this study. Data sharing is not applicable to this article.

## Abbreviations

The following abbreviations are used in this manuscript:

5-ASA	5-aminosalicylic acid
ALP	Alkaline phosphatase
ALT	Alanine transaminase
AST	Aspartate transaminase
Ca^2+^	Intracellular calcium
cAMP	Cyclic adenosine monophosphate
CAT	Catalase
CBWR	Colon weight-to-body weight ratio
CD	Crohn’s disease
COX-2	Cyclooxygenase-2
cPLA2	Cytosolic phospholipase A2
CRD	Colorectal distension
DASR	Disease activity score
DSS	Dextran sodium sulfate
EMG	Electromyography
eNOS	Endothelial nitric oxide synthase
Gi/o proteins	G inhibitory/other proteins
Gq	Gq-protein coupled receptor
Gs protein	Stimulatory G protein
GSH	Reduced glutathione
GST	Glutathione S-transferase
H1R	Histamine H1 receptors
H2R	Histamine H2 receptors
H2RA	Histamine H2 receptor antagonist
H3R	Histamine H3 receptors
H4R	Histamine H4 receptors
HDC	Histidine decarboxylase
IBD	Inflammatory bowel disease
IFN-γ	Interferon gamma
IL-17	Interleukin 17
IL-1β	Interleukin 1β
IL-6	Interleukin 6
IP3	Inositol 1,4,5-trisphosphate
MC	Microscopic colitis
MDA	Malondialdehyde
MPO	Myeloperoxidase
MTX	Methotrexate
NADPH	Nicotinamide adenine dinucleotide phosphate (reduced form)
NAFLD	Non-alcoholic fatty liver disease
NF-κB	Nuclear factor kappa- B
Nrf2	Nuclear factor erythroid 2-related factor 2 (Nrf2)
NSAID	Non-steroidal anti-inflammatory drug
PGE2	Prostaglandin E2
p-IκBα	Phosphorylated inhibitor of κb alpha
pKi	Negative logarithm of the inhibition constant (Ki)
PLC	Phospholipase C
PMSI	Partial mayo scoring index
p-p65	Phosphorylated p65 subunit of NF-κb
PPIs	Proton pump inhibitors
PSC	Primary sclerosing cholangitis
PUFAs	Polyunsaturated fatty acids
RAMH	(R)-α-methylhistamine
ROS	Reactive oxygen species
SOD	Superoxide dismutase
STAT3	Signal transducer and activator of transcription 3
TAC	Total antioxidant capacity
Th1	T helper 1 cells
Th17	T helper 17 cells
TNBS	2,4,6-trinitrobenzene sulfonic acid
TNF-α	Tumor necrosis factor α
TOS	Total oxidative status
UC	Ulcerative colitis
VMRs	Visceromotor responses
